# Supplementary material for: Integrative analysis of non-small cell lung cancer patient-derived xenografts identifies distinct proteotypes associated with patient outcomes
Source: Nat Commun. 2022 Apr 5;13:1811. doi: 10.1038/s41467-022-29444-9 (PMC8983714; doi:10.1038/s41467-022-29444-9)
Supplement: Supplementary file 1 — Supplementary Information [file 41467_2022_29444_MOESM1_ESM.pdf]

SUPPLEMENTARY INFORMATION

A

Subtype	TCGA LUAD (n=533)	UHN PDX (n=51)
TRU	39%	37%
Proximal Proliferative	27%	33%
Proximal Inflammatory	34%	29%

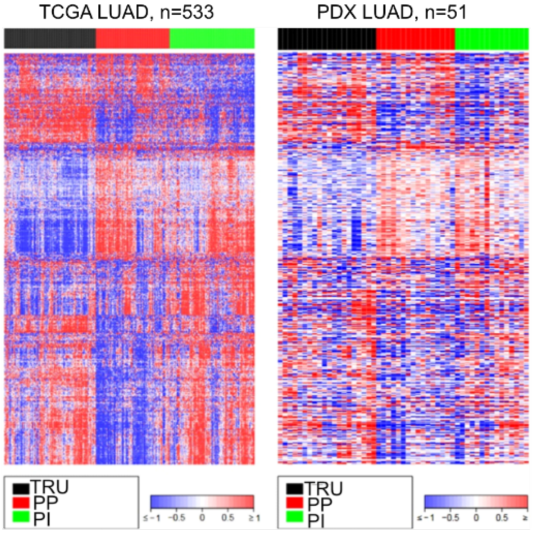

C

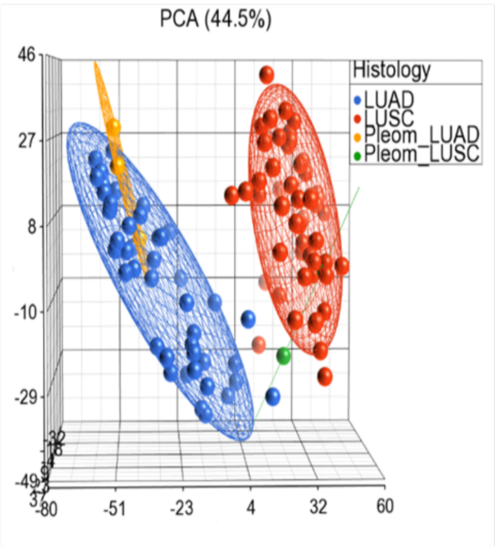

B

Subtype	TCGA LUSC (n=501)	UHN PDX (n=59)
Primitive	14%	15%
Classical	34%	36%
Secretory	24%	32%
Basal	28%	17%

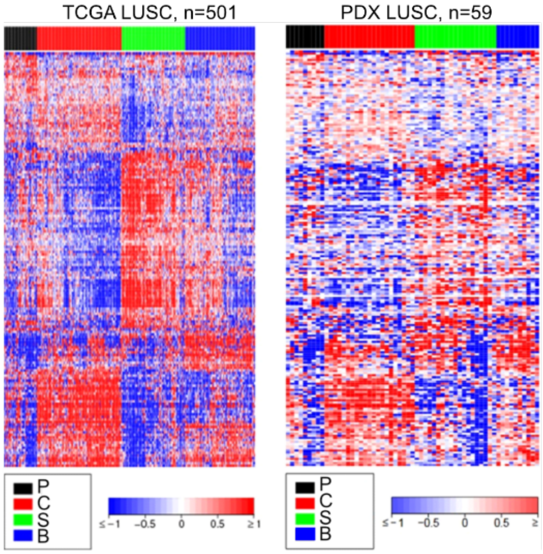

Supplementary Figure 1-PDX transcriptomic subtype distribution represent primaries and separation of LUAD from LUSC by methylome-A) Comparison of the distribution of LUAD subtypes between the entire TCGA cohort (https://portal.gdc.cancer.gov/) and PDX cohort reported in this manuscript. (TRU=Terminal Respiratory Unit; PP=Proximal Proliferative; PI=Proximal Inflammatory). **B)** Comparison of the distribution of LUSC subtypes between the entire TCGA cohort (https://portal.gdc.cancer.gov/) and PDX cohort reported in this manuscript. (P=Primitive; C=Classical; S=secretory; B=Basal). **C)** The 3D PCA plot with ellipses concentration was generated using 4,000 most variable probes, showing distinct separation of the LUAD and LUSC PDX tumor samples.

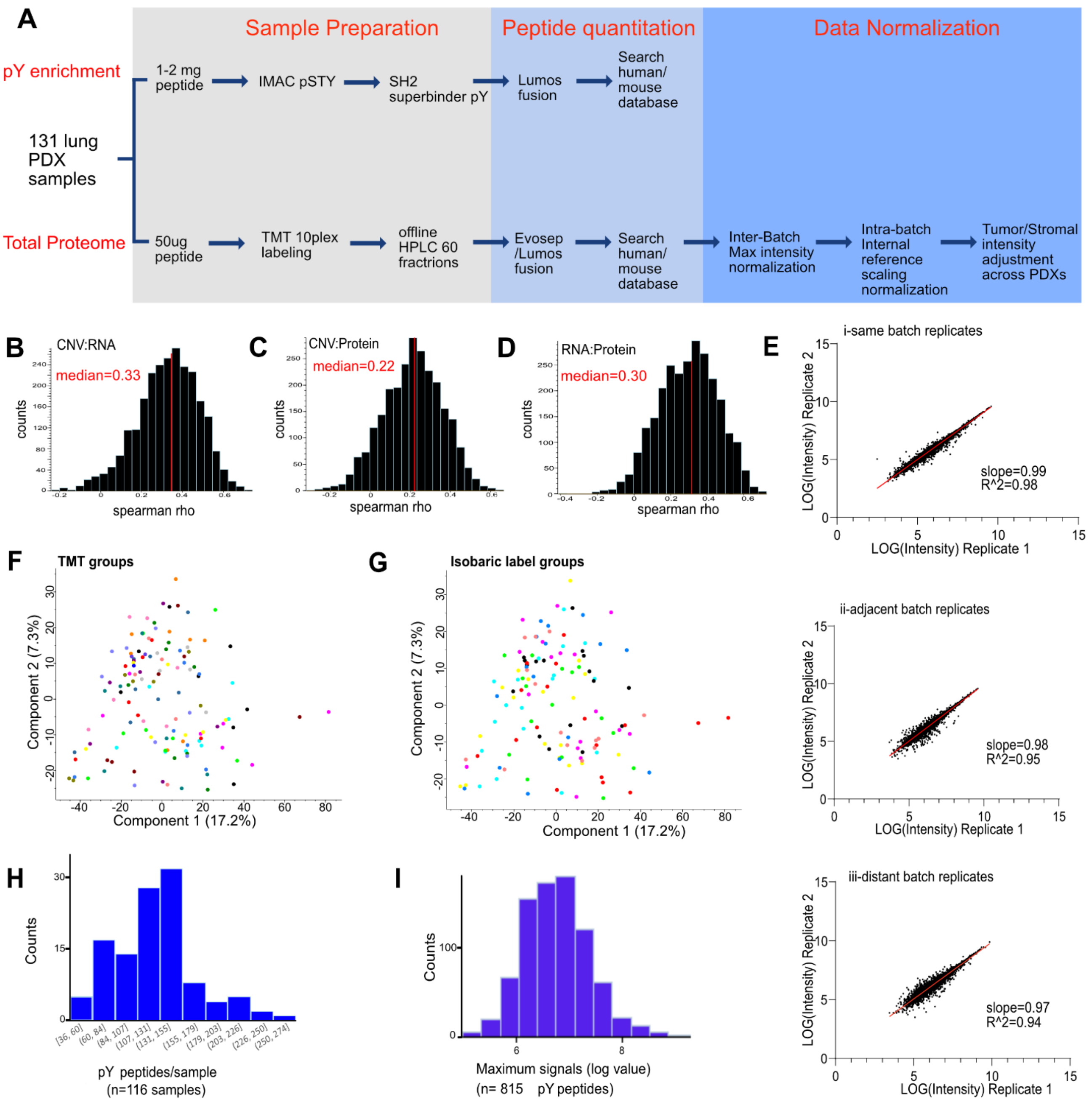

Supplementary Figure 2-Proteome and pY-proteome workflow, data quality assurance, and correlations across molecules-A) Proteome and phosphoproteome sample preparation, peptide quantification and data normalization workflow. **B)** CNV-RNA pairwise spearman correlation across all patients shows distribution of spearman rho for all genes identified in all patients at the proteome level with median of 0.33. See also Supplementary data 1. **C)** CNV-Protein pairwise spearman correlation across all patients shows distribution of spearman rho for all genes identified in all patients at the proteome level with median of 0.22. See also Supplementary data 1. **D)** RNA-Protein pairwise spearman correlation across all patients shows distribution of spearman rho for all genes identified in all patients at the proteome level with median of 0.3. See also Supplementary data 1. **E)** linear relationship between technical repeats of the i-same ii-adjacent and iii-distant batch. **F)** PCA analysis of all cases, each color represents a different TMT group. Figure shows no TMT batch affect is seen. **G)** PCA analysis of all cases, each color represents a different isobaric label. Figure shows no isobaric label affect is seen **H)** Distribution of number of pY peptides per sample show almost a normal distribution **I)** Distribution of maximum signals show almost a normal distribution.

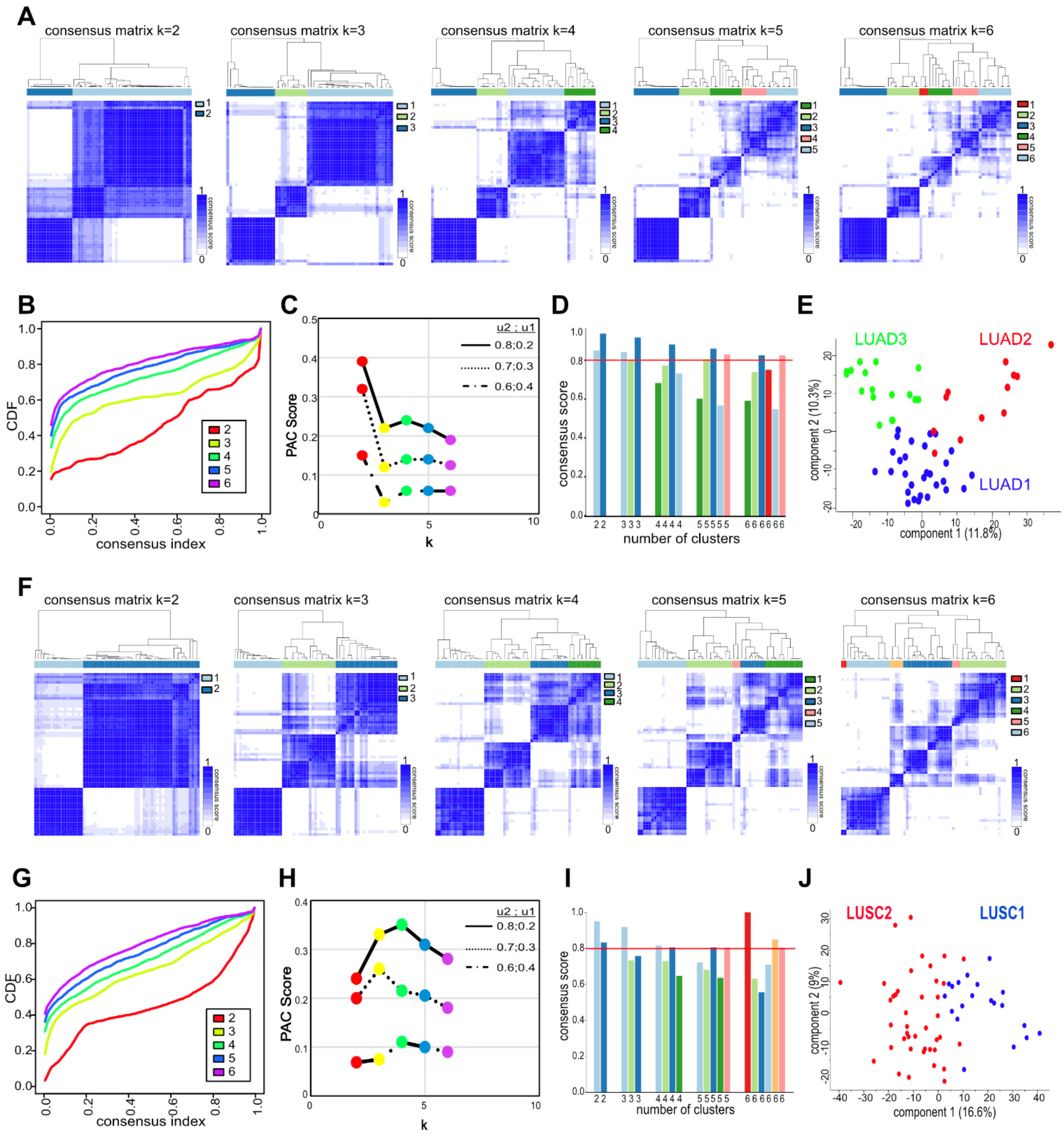

Supplementary Figure 3- Identification of 3 LUAD and 2 LUSC proteotypes-A) Heatmap of consensus matrices for k=2-6 for LUAD. **B)** Cumulative density frequency (CDF) plot for K=2-6 for LUAD. **C)** Proportion of ambiguous clustering (PAC) scores for k=2-6 for LUAD show optimal cluster at k=3. **D)** Cluster consensus plot for k=2-6 for LUAD. **E)** PCA of LUAD samples segregated into the 3 identified groups. **F)** Heatmap of consensus matrices for k=2-6 for LUSC. **G)** CDF plot for K=2-6 for LUSC. **H)** PAC scores for k=2-6 for LUSC show optimal cluster at k=2. **I)** Cluster consensus plot at k=2-6 for LUSC. **J)** PCA of LUSC samples segregated into the 2 identified groups.

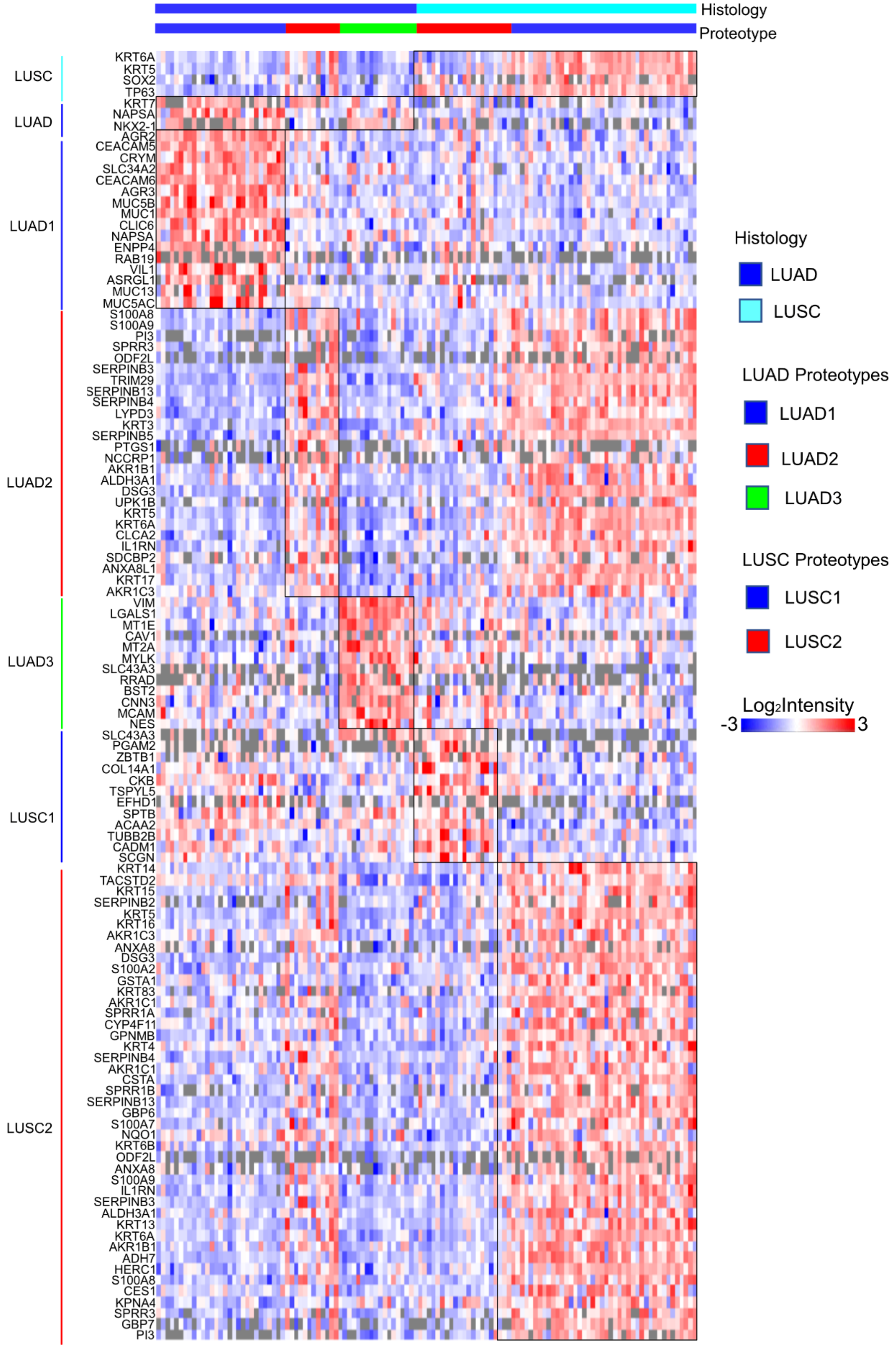

Supplementary Figure 4-Proteotype markers: Protein expression of histological subtypes and proteotype markers across PDXs-The proteome expression of biomarkers across PDXs are shown. From the top markers are for: LUSC and LUAD histological subtypes, followed by proteotype markers for LUAD1, LUAD2, LUAD3, LUSC1, and LUSC2.

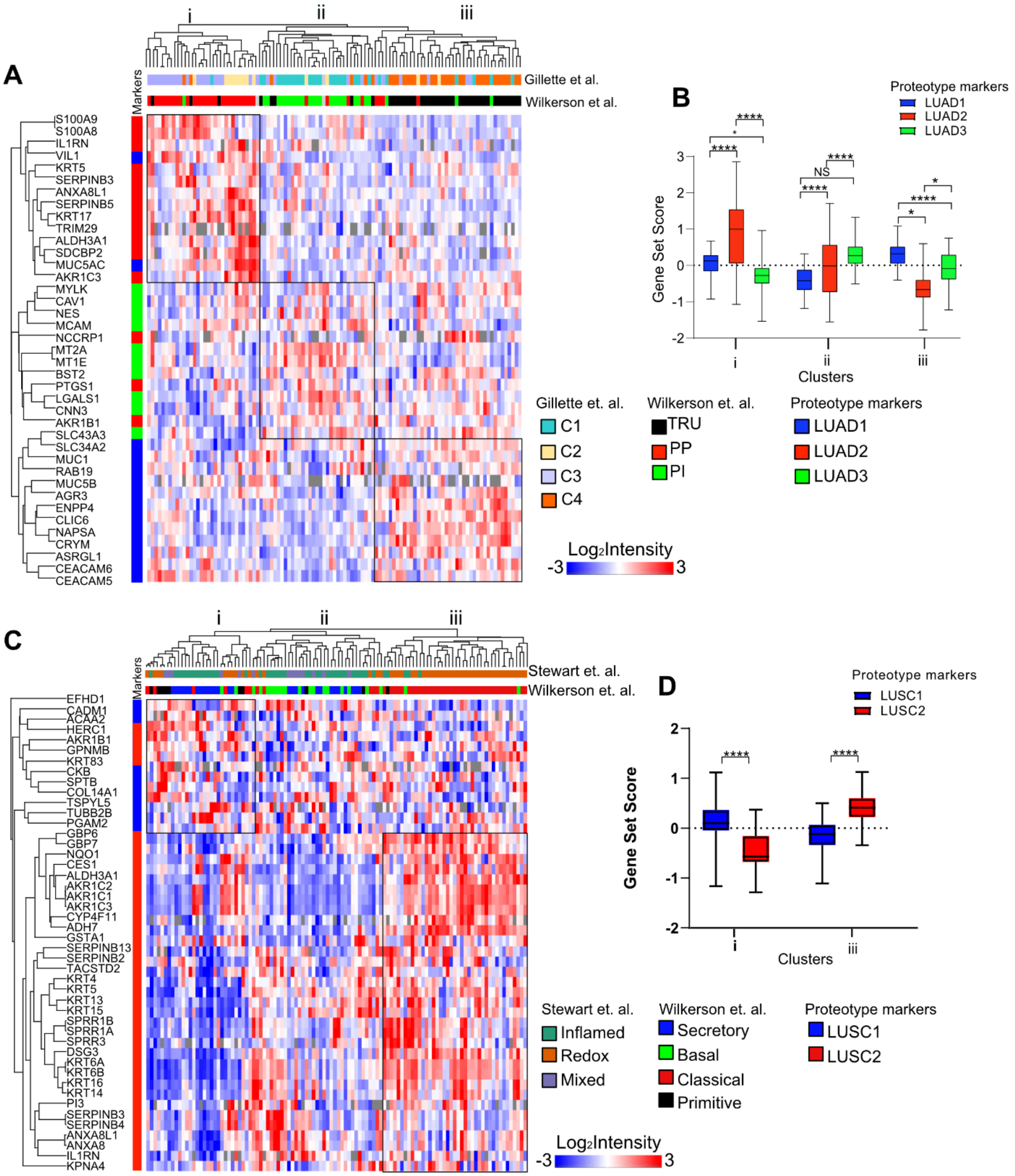

Supplementary Figure 5-LUAD and LUSC proteotype marker validation in independent primary cohorts- A) Unsupervised hierarchical clustering of an unrelated 107 LUAD tumors (Gillette et. al., 2020) with LUAD proteotype markers identified in this study. **B)** The expression of LUAD1, 2 and 3 markers are significantly higher in cluster iii (n=42), i (n=32) and ii (n=33) respectively* as determined by 2way ANOVA (p-value=4.54E-25). **C)** Unsupervised hierarchical clustering of an unrelated 108 LUSC tumor (Stewart et. al., 2019) with LUSC proteotype markers identified in this study. **D)** The expression of LUSC1 and 2 markers are significantly higher in cluster i (n=30), and iii (n=41) respectively* as determined by 2way ANOVA (p-value=2.8E-15)

*One-sided Tukey's adjusted for multiple comparison p-value= ****<0.0001, **<0.005, *<0.05. Box plots showing median at centre line, whiskers showing minimum and maximum values and box showing the 25-75% percentile.

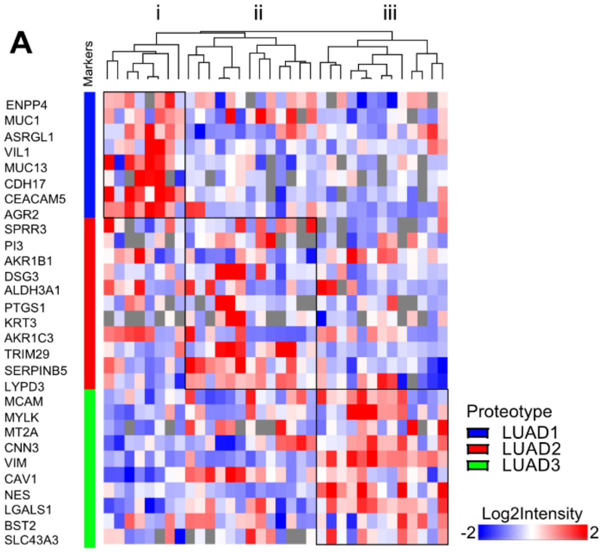

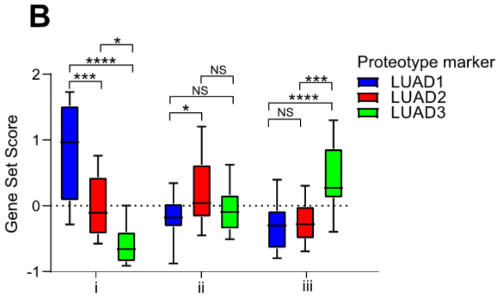

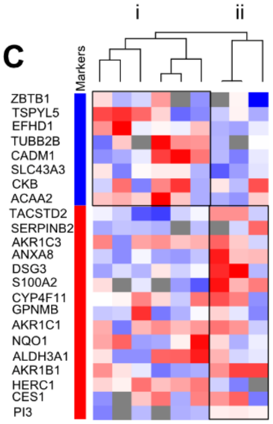

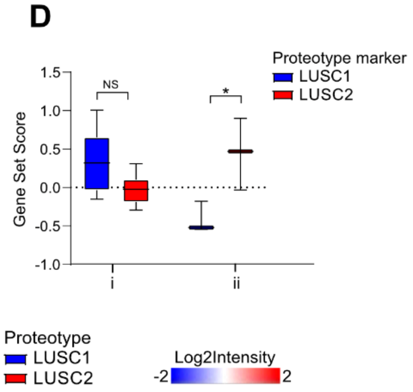

E

	Protein	Effect Size	Significance (Qvalue)	#Cell Lines	Assay
LUAD1	FRS3	0.10872	0.02167	26	RNAi
	UBAP2	0.06573	0.01994	27	CRISPR
	FCRLB	0.0607	0.02604	27	CRISPR
	PDF	0.05958	0.03664	27	CRISPR
	KRT33B	0.05954	0.02883	27	CRISPR
	SPDEF	0.05885	0.04032	27	CRISPR
	LBR	0.05851	0.04339	27	CRISPR
	FGFBP2	0.05783	0.04481	27	CRISPR
	MAOA	0.05707	0.04622	27	CRISPR
	UNC45B	0.05688	0.04751	27	CRISPR
LUAD2	TAS1R1	0.10811	0.00296	27	CRISPR
	WLS	0.10462	0.00485	17	RNAi
	CST11	0.09674	0.00732	26	RNAi
	PROB1	0.09353	0.00647	27	CRISPR
	CCDC116	0.06297	0.03949	17	RNAi
	HLX	0.0601	0.0384	27	CRISPR
	REEP1	0.05146	0.02882	27	CRISPR
	OPN5	0.04579	0.02994	32	RNAi
	MAPK6	0.03972	0.01894	32	RNAi
	SETD7	0.03956	0.04662	32	RNAi
LUAD3	CST7	0.07425	0.02823	26	RNAi
	OAS1	0.05692	0.00947	32	RNAi
	PDK4	0.04874	0.0387	32	RNAi
LUSC1	HOOK3	0.20724	0.00001	9	CRISPR
	GMEB2	0.15071	0.00027	9	CRISPR
	ST6GALNAC6	0.15016	0.00014	9	CRISPR
	ZNF837	0.14805	0.00056	9	CRISPR
	ZNF865	0.14303	0.00045	9	CRISPR
	TEC	0.14099	0.00046	9	CRISPR
	TANC2	0.13875	0.00029	9	CRISPR
	ZNF606	0.1169	0.00043	9	CRISPR
	TAAR2	0.10112	0.00029	9	CRISPR
	S100A8	0.17876	0.00063	9	CRISPR
LUSC2	ASF1B	0.33021	0.00033	9	CRISPR
	AKTIP	0.26264	0.00027	8	RNAi
	APRT	0.23532	0.00059	8	RNAi
	H4C5	0.20184	0.00057	9	CRISPR
	FANCB	0.19153	0.00035	9	CRISPR
	PFN2	0.1876	0.0005	9	CRISPR
	ARAP1	0.18163	0.00027	9	CRISPR
	GPM6B	0.18063	0.00011	9	CRISPR
	PDHA2	0.1746	0.00021	9	CRISPR
	IP6K1	0.16158	0.00034	9	CRISPR

F

	label	Effect Size	QValue	#Cell Lines
LUAD1	stattic (BRD:BRD-K17061841-001-03-5) 0.625µM	0.37915	0.0348	23
LUAD2	oxiconazole (BRD:BRD-K56104152-008-04-1)	0.32352	0.01822	29
	U-0124 (BRD:BRD-K63169195-001-01-5)	0.31799	0.02235	29
	LY255283 (BRD:BRD-K92492521-001-02-9)	0.29259	0.03272	29
LUAD3	ZM-306416 (BRD:BRD-K41337261-001-03-0) 0.0390625µM	0.27913	0.00554	26
	halcinonide (BRD:BRD-A88138582-001-04-8) 10µM	0.27899	0.01802	26
	ixabepilone (BRD:BRD-K03601870-001-01-2) 0.00976562µM	0.26887	0.00581	28
LUSC1	echinomycin (BRD:BRD-A68969091-001-12-7)	2.14323	0.00257	7
	docetaxel (BRD:BRD-K30577245-341-01-9)	2.11585	0.00284	7
	pyrithione-zinc (BRD:BRD-K16136380-001-01-7)	1.71951	0.00313	7
LUSC2	gossypol (BRD:BRD-K19295594-015-14-3) 9.1496µM	2.46768	0.00286	7
	mefexamide (BRD:BRD-K20655524-003-12-0) 0.15625µM	1.56048	0.0034	6
	dacinostat (BRD:BRD-K56957086-001-06-3) 2.5µM	1.14149	0.00587	6

Supplementary Figure 6-Proteotyping DepMap NSCLC cell lines with markers reveals proteotype-specific sensitivities and validate study's target predictions. A) LUAD markers used to cluster of 34 unrelated LUAD DEPMAP cell lines identifies 3 cluster i, ii, and iii with respectively higher LUAD1,2 and 3 expressions. **B)** The expression of LUAD1, 2 and 3 markers are significantly higher in cluster i (n=8), ii (n=13) and iii (n=13) respectively* determined by 2way ANOVA (p-value=2.68E-11). **C)** LUSC markers used to cluster 9 unrelated LUSC DEPMAP cell lines identifies 2 cluster i and ii with respectively higher LUSC1 and 2 expressions. **D)** The expression of LUSC1 markers is higher in cluster i (n=6) and LUSC2 markers are significantly higher in cluster ii (n=3) *determined by 2way ANOVA (p-value=0.0031). **E)** Top 10 sensitivities with the highest difference in effect size on proliferation for each proteotype in DepMap cells. **F)** Top 3 most significant specific drug sensitivities for each proteotype in DepMap cells.

*One-sided Tukey's adjusted for multiple comparison p-value= ****<0.0001, **<0.005, *<0.05. Box plots showing median at centre line, whiskers showing minimum and maximum values and box showing the 25-75% percentile.

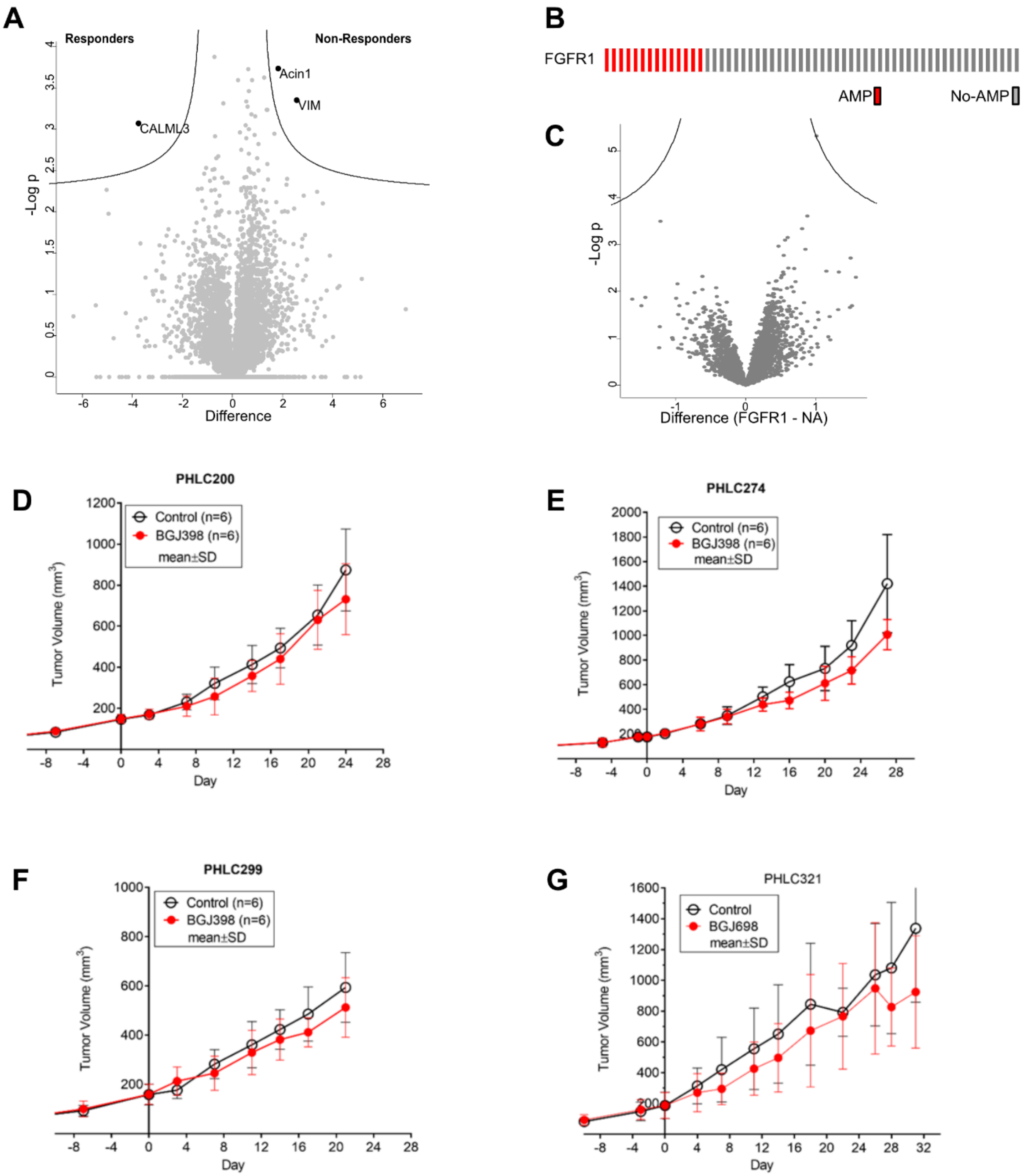

Supplementary Figure 7-FGFR1 inhibitor, BGJ398 Response curves in FGFR1 amplified PDX models-A) Proteome differences between EGFR TKI non-responders (PHLC148,-164) vs responders (PHLC137, -192) using a two-sided t-test (FDR<0.05). **B)** Proportion of FGFR1 amplified cases among LUSC is 17 of 58 (29%). **C)** No proteins are significantly different between AMP and no-AMP cases (two-sided t-test, FDR<0.05). **D-G)** FGFR1 inhibitor BGJ398 did not show a significant response compared to control in four FGFR1-amplified PDX models (linear mixed effects models test, p>0.1): **D)** PHLC-200, **E)** PHLC-274, **F)** PHLC-299, and **G)** PHLC-321. All error bars are shown as mean±SD.

Supplementary Table 1. Breakdown of patient tumors used for engraftment.

	Resection	EBUS	Total
NSCLC patients with tumor implanted	443	57	500
Non-engraftment	237	44	281
Formed lymphoma at P0	56	1	57
Engrafted Carcinoma at P0	150	13	163
Unstable engraftment (<P2)	25	1	26
Stable PDX growth (>P2)	125	12	137

Supplementary Table 2. Patient Summary

Covariate	n=137
**age at procedure**	
Mean (sd)	67.8 (10.3)
Median (Min,Max)	67.1 (44.1,90.2)
**sex**	
M	87 (64)
F	50 (36)
**hist**	
LUSC	65 (47)
LUAD	58 (42)
LCNEC	6 (4)
Pleom LUSC	2 (1)
Pleom LAUD	3 (2)
LCC	2 (1)
ADSQ	1 (1)
**smoking**	
Ever	123 (91)
Never	12 (9)
Missing	**2**
**pathStage**	
1	40 (30)
2	49 (37)
3	33 (25)
4	12 (9)
Missing	**3**
**adj ch**	
Y	49 (36)
N	88 (64)
**adj rad**	
Y	17 (12)
N	119 (88)
Missing	**1**
**resection**	
Y	125 (100)
Missing	**12**

Supplementary Table 3. Proteotypes' associations (Fisher's exact test) with clinical and genomic attributes.

	LUAD1	LUAD2	LUAD3	LUSC1	LUSC2
Stage	p=0.0177				
Smoking Status					
Sex					
Transcriptome Subtype			p=0.009	p=0.004	
Methylome Subtype		p=0.0015	p=0.0053		

early
not enriched
PI
primitive
intermediate
low

Supplementary Table 4. Univariate survival using Cox-proportional hazard model for LUAD and LUSC proteotypes.

Covariate	HR(95%CI)	p-value	Global p-value
**LUAD**			0.1
LUAD2 vs LUAD1	1.28 (0.41, 4.03)	0.67	
LUAD3 vs LUAD1	2.6 (1.07, 6.3)	0.034	
**LUSC**			0.049
LUSC2 vs LUSC1	3.48 (1,12.06)		

Supplementary Table 5. Multivariate survival with stage using Cox-proportional hazard model for LUAD proteotypes

Covariate	HR (95% CI)	p-value	Global p-value
**pathStage12 34**			0.034
1/2	reference		
3/4	2.59 (1.08,6.25)		
**cluster**			0.5
1	reference		
2	1.04 (0.32,3.25)	0.94	
3	1.73 (0.66,4.55)	0.27	

Supplementary Table 6. Multivariate survival with stage using Cox-proportional hazard model for LUSC proteotypes

Covariate	HR (95% CI)	p-value	Global p-value
**pathStage12 34**			0.074
1/2	reference		
3/4	2.55 (0.91, 7.15)		
**cluster**			0.079
1	reference		
2	2.71 (0.74, 9.87)		

Supplementary Table 7. Validation cluster associations (Fisher's exact test) with original study's subtypes.

<table>
<tr><th></th><th></th><th>iii/LUAD1</th><th>i/LUAD2</th><th>ii/LUAD3</th></tr>
<tr><td rowspan="4">Gillettel</td><td>C1</td><td></td><td></td><td>p=4.9E-07</td></tr>
<tr><td>C2</td><td rowspan="2"></td><td rowspan="2">p=2.9E-06</td><td rowspan="2"></td></tr>
<tr><td>C3</td></tr>
<tr><td>C4</td><td>p=5.7E-08</td><td></td><td></td></tr>
</table>

☐not enriched

<table>
<tr><th></th><th></th><th>i/lusc1</th><th>iii/lusc2</th></tr>
<tr><td rowspan="2">Stewart</td><td>inflamed</td><td>p=0.0009</td><td></td></tr>
<tr><td>Redox</td><td></td><td>p=2.5E-11</td></tr>
</table>

☐not enriched
