# Supplementary material for: Integrative analysis of non-small cell lung cancer patient-derived xenografts identifies distinct proteotypes associated with patient outcomes
Source: Nat Commun. 2022 Apr 5;13:1811. doi: 10.1038/s41467-022-29444-9 (PMC8983714; doi:10.1038/s41467-022-29444-9)
Supplement: Supplementary file 3 — Description of Additional Supplementary Files [file 41467_2022_29444_MOESM3_ESM.pdf]

Description of Additional Supplementary Files

File Name: Supplementary Data 1
Description: Clinical information, Omic-data, and cross platform correlations

File Name: Supplementary Data 2
Description: -Differential Proteome and pathways

File Name: Supplementary Data 3
Description: Differential pY and pathways

File Name: Supplementary Data 4
Description: DepMap proteotyping and sensitivities
