# Supplementary material for: Integrative analysis of non-small cell lung cancer patient-derived xenografts identifies distinct proteotypes associated with patient outcomes
Source: Nat Commun. 2022 Apr 5;13:1811. doi: 10.1038/s41467-022-29444-9 (PMC8983714; doi:10.1038/s41467-022-29444-9)
Supplement: Supplementary file 8 — Reporting Summary [file 41467_2022_29444_MOESM8_ESM.pdf]

nature research

Corresponding author(s): Michael F. Moran and Ming-Sound Tsao

Last updated by author(s): 22-03-14

Reporting Summary

Nature Research wishes to improve the reproducibility of the work that we publish. This form provides structure for consistency and transparency in reporting. For further information on Nature Research policies, see our Editorial Policies and the Editorial Policy Checklist.

Statistics

For all statistical analyses, confirm that the following items are present in the figure legend, table legend, main text, or Methods section.

n/a	Confirmed	
☐	☒	The exact sample size (*n*) for each experimental group/condition, given as a discrete number and unit of measurement
☐	☒	A statement on whether measurements were taken from distinct samples or whether the same sample was measured repeatedly
☐	☒	The statistical test(s) used AND whether they are one- or two-sided<br>*Only common tests should be described solely by name; describe more complex techniques in the Methods section.*
☐	☒	A description of all covariates tested
☐	☒	A description of any assumptions or corrections, such as tests of normality and adjustment for multiple comparisons
☐	☒	A full description of the statistical parameters including central tendency (e.g. means) or other basic estimates (e.g. regression coefficient) AND variation (e.g. standard deviation) or associated estimates of uncertainty (e.g. confidence intervals)
☐	☒	For null hypothesis testing, the test statistic (e.g. *F*, *t*, *r*) with confidence intervals, effect sizes, degrees of freedom and *P* value noted<br>*Give P values as exact values whenever suitable.*
☒	☐	For Bayesian analysis, information on the choice of priors and Markov chain Monte Carlo settings
☐	☒	For hierarchical and complex designs, identification of the appropriate level for tests and full reporting of outcomes
☐	☒	Estimates of effect sizes (e.g. Cohen's *d*, Pearson's *r*), indicating how they were calculated

Our web collection on statistics for biologists contains articles on many of the points above.

Software and code

Policy information about availability of computer code

Data collection	The study does not utilize software or code for data collection.
Data analysis	No custom code was used to analyze the data in this study. A list of commercial and open source codes used in the study are listed below seperated by comma. Xenome (V1.0.1), Burrows-Wheeler Aligner (v0.7.12), Genome Analysis Toolkit (v3.4), samtools (v1.2), Picard (v1.140), minfi (v1.6.0), IlluminaHumanMethylation450kmanifest (v0.4.0), sva(v3.20.0), Genome Studio Illumina, lumi (v2.24.0), ASCAT(v2.5), MuTect (v1.0), VarScan (v2.3.8), ANNOVAR(annovar/20160201), GISTIC(v.2.0.23), MaxQuant (1.6.2.10), Partek Genome Suite Software(7.0), ConsensusClusterPlus(1.36.0), Perseus(1.6.5.0), Ingenuity Pathway Analysis (2020-2021)

For manuscripts utilizing custom algorithms or software that are central to the research but not yet described in published literature, software must be made available to editors and reviewers. We strongly encourage code deposition in a community repository (e.g. GitHub). See the Nature Research guidelines for submitting code & software for further information.

Data

Policy information about availability of data

All manuscripts must include a data availability statement. This statement should provide the following information, where applicable:

- Accession codes, unique identifiers, or web links for publicly available datasets
- A list of figures that have associated raw data
- A description of any restrictions on data availability

PDX models are listed in the open global catalogue of PDX models at the PDXFinder repository (pdxfinder.org). PDX models generated in this study are available for research use with institutional material transfer agreement. The mass spectrometry proteomics and phosphotyrosine data generated in this study have been deposited to the ProteomeXchange Consortium via the PRIDE partner repository 79 with the dataset identifier PXD016579[http://www.ebi.ac.uk/pride/archive/projects/PXD016579] for total proteome and PXD016674[http://www.ebi.ac.uk/pride/archive/projects/[http://www.ebi.ac.uk/pride/archive/projects/PXD016674]

for phosphotyrosine. The gene expression data generated in this study has been deposited to GEO with the dataset identifier of GSE166999 [https://www.ncbi.nlm.nih.gov/geo/query/acc.cgi?acc=GSE166999]. DNA methylation data generated in this study has been deposited to EBI with the dataset identifier of E-MTAB-10156 proteome[https://www.ebi.ac.uk/arrayexpress/experiments/E-MTAB-10156]. The raw whole exome sequencing data generated in this study are deposited to the European Genome-phenome Archive (https://ega-archive.org/) under Dataset ID: EGAD00001008601. The data are available under restricted access due to laws to protect the privacy of patients in alignment with University Health Network (UHN) Review Ethics Board (REB) approvals and individual patient informed consent forms. Access to the data can be obtained by qualified researchers as part of an academic or industry collaboration. Requests should include a research proposal indicating the intended use of data and planned analyses. Requests will be reviewed typically within two weeks by the UHN Data Access Committee (DAC) and should be made by using the DAC ID: EGAC00001000912. There are no time constraints on data access. Minimal essential PDX model characteristics have been deposited to the PDX Finder repository (https://www.pdxfinder.org/source/PMLB/). The publicly available DepMap sensitivity data28,29 used in this study are available through DepMap portal [https://depmap.org/portal/download/]. The publicly available data generated by Gillette et al13 is available via CPTAC data portal [https://cptac-data-portal.georgetown.edu/cptac/s/S056]. The publicly available data generated by Stewart et al15. was deposited to the ProteomeXchange Consortium via the PRIDE partner repository 79 with the dataset identifier PXD010429 [https://doi.org/10.6019/PXD010429]. The publicly available database dbSNP (version 138 flagged) can be found at https://ftp.ncbi.nlm.nih.gov/snp/, ExAC03 can be found at https://gnomad.broadinstitute.org/ and ESP6500 can be found at https://evs.gs.washington.edu/EVS/. The remaining data are available within the Article, Supplementary Information or Source Data file.

Field-specific reporting

Please select the one below that is the best fit for your research. If you are not sure, read the appropriate sections before making your selection.

- [x] Life sciences
- [ ] Behavioural & social sciences
- [ ] Ecological, evolutionary & environmental sciences

For a reference copy of the document with all sections, see nature.com/documents/nr-reporting-summary-flat.pdf

Life sciences study design

All studies must disclose on these points even when the disclosure is negative.

Sample size	The number of NSCLC cases was determined by specimens that had excess tissues for research between 2005 and 2014. The sample size (137) was the subset of NSCLC primary tumors from 500 tumors that could establish a stable patient-derived xenograft in NOD-SCID mice.
Data exclusions	No data was excluded from analysis
Replication	The proteome samples were analyzed using 10plex-Tandem mass tags, in which two channels were controls. The same control sample was used in each 10-plex sample set. In total 34 control replicates were run in this study. This control was composed of a mixture of all 137 tumors. These 34 control replicates are used to assess the quality of proteome data per experimental group and to normalize the data. All attempts of replication were successful. No other attempts at replication other than what is included here were attempted. Other genomic platforms included in the study do not include replication as part of the study design. PDX drug studies were performed with mouse replicates (5-6) per group per study.
Randomization	The selection of PDX tumors for placement into experimental groups for all proteome and genome profiling were completely randomized by assigning alias names which did not reveal identity and attributes of the samples at the time of sample processing. For drug studies, mouse replicates in PDX studies were stratified randomly to each group, to equally distribute tumor volumes and mouse body weights.
Blinding	To avoid bias in analyses, investigators analyzing the proteome and phosphoproteome data were blinded to the clinical attributes of the samples. The subtypes once determined were assessed for survival comparison by an independent group. The PDX studies were not performed in a blinded manner, drug anti-tumor effects and drug toxicity will be obvious to the staff. The vehicle and the drug treated mice were housed together, and to minimize any biases, the staff systematically took measurements and administer the agents, vehicle or drug as designated per mouse by their sequential order in the housing.

Reporting for specific materials, systems and methods

We require information from authors about some types of materials, experimental systems and methods used in many studies. Here, indicate whether each material, system or method listed is relevant to your study. If you are not sure if a list item applies to your research, read the appropriate section before selecting a response.

Materials & experimental systems

n/a	Involved in the study	
x		Antibodies
x		Eukaryotic cell lines
x		Palaeontology and archaeology
	x	Animals and other organisms
	x	Human research participants
x		Clinical data
x		Dual use research of concern

Methods

n/a	Involved in the study	
x		ChIP-seq
x		Flow cytometry
x		MRI-based neuroimaging

Animals and other organisms

Policy information about studies involving animals; ARRIVE guidelines recommended for reporting animal research

Laboratory animals	Male, NOD SCID mice (4-6 weeks) were used as hosts.
Wild animals	The study did not involve wild animals.
Field-collected samples	The study did not involve samples collected from the field.
Ethics oversight	The University Health Network Animal Care (AUP603) for model establishment and AUP743 for drug study Committees approved this study protocol. Animal care followed the guidelines of UHN Research Institutes' policies and the guidelines of the Canadian Council on Animal Care, and consistent with ARRIVE guidelines for study design.

Note that full information on the approval of the study protocol must also be provided in the manuscript.

Human research participants

Policy information about studies involving human research participants

Population characteristics	All clinical attributes of patients such as age, sex, smoking status, etc. are summarized in Table S1 and S2 and detailed in Table S8.
Recruitment	A total of 500 patients were included in this study with the patient's informed consent. No participant compensation was provided.
Ethics oversight	Tumor specimens were collected at the Toronto General Hospital (TGH-UHN) between 2005 and 2014 using The University Health Network (UHN) Human Research Ethics protocol 09-0510-T. Human research followed the guidelines of Canada Tri-Council Policy Statement, in accordance with Declaration of Helsinki (www.pre.ethics.gc.ca.).

Note that full information on the approval of the study protocol must also be provided in the manuscript.
